# High-Performance Self-Powered Photodetector Enabled by Te-Doped GeH Nanostructures Engineering

**DOI:** 10.3390/s25082530

**Published:** 2025-04-17

**Authors:** Junting Zhang, Jiexin Chen, Shuojia Zheng, Da Zhang, Shaojuan Luo, Huixia Luo

**Affiliations:** 1 School of Chemical Engineering and Light Industry, Guangdong University of Technology, Guangzhou 510006, China; 2 State Key Laboratory of Optoelectronic Materials and Technologies, Sun Yat-sen University, Guangzhou 510275, China; 3 Jieyang Branch of Chemistry and Chemical Engineering Guangdong Laboratory, Jieyang 515200, China

**Keywords:** Te-GeH nanostructures, photodetector, photoelectrochemical, self-powered

## Abstract

**Highlights:**

**What are the main findings?**

- A facile chemical strategy was developed for the synthesis of two-dimensional Te-doped GeH nanostructures with atomic-level precision and structural integrity.
- The Te-GeH-based photoelectrochemical (PEC) photodetectors exhibit high responsivity (708.5 µA/W) and ultrafast response speeds (92 ms rise/526 ms decay) under zero-bias conditions, demonstrating excellent broadband photoresponse.

**What is the implication of the main finding?**

- The introduction of Te atoms enables effective modulation of the electronic structure in the GeH system. Owing to the comparable atomic radius between Te and Ge, efficient doping is achieved without disrupting the host lattice. In addition, the high electronegativity of Te induces electron redistribution and facilitates the formation of a built-in electric field, thereby optimizing charge separation and transport behavior. This approach provides a novel pathway for the functional design of Zintl-phase-derived two-dimensional materials, with broad implications for advanced optoelectronic applications.
- This doping strategy significantly enhances the photoelectrochemical performance of GeH-based photodetectors and enables the scalable fabrication of 2D nanomaterials with tunable optoelectronic properties. The resulting Te-GeH nanostructures offer improved carrier dynamics and reduced recombination, serving as a promising material platform for next-generation self-powered broadband photodetectors.

**Abstract:**

Two-dimensional (2D) Xenes, including graphene where X represents C, Si, Ge, and Te, represent a groundbreaking class of materials renowned for their extraordinary electrical transport properties, robust photoresponse, and Quantum Spin Hall effects. With the growing interest in 2D materials, research on germanene-based systems remains relatively underexplored despite their potential for tailored optoelectronic functionalities. Herein, we demonstrate a facile and rapid chemical synthesis of tellurium-doped germanene hydride (Te-GeH) nanostructures (NSs), achieving precise atomic-scale control. The 2D Te-GeH NSs exhibit a broadband optical absorption spanning ultraviolet (UV) to visible light (VIS), which is a critical feature for multifunctional photodetection. Leveraging this property, we engineer photoelectrochemical (PEC) photodetectors via a simple drop-casting technique. The devices deliver excellent performance, including a high responsivity of 708.5 µA/W, ultrafast response speeds (92 ms rise, 526 ms decay), and a wide operational bandwidth. Remarkably, the detectors operate efficiently at zero-bias voltage, outperforming most existing 2D-material-based PEC systems, and function as self-powered broadband photodetectors. This work not only advances the understanding of germanene derivatives but also unlocks their potential for next-generation optoelectronics, such as energy-efficient sensors and adaptive optical networks.

## 1. Introduction

Photodetectors have attracted notable attention due to their wide-ranging applications in multiple fields, such as optical communication, imaging technologies, and missile guidance, which are relevant to both civilian and defense sectors [1,2,3,4]. Recently, photoelectrochemical (PEC) photodetectors have garnered significant attention as promising candidates for next-generation optoelectronic devices due to their simple fabrication processes and self-powered mechanisms [5].

The performance of photodetectors is strongly influenced by the properties of the photoelectric materials used. As the demand for more functional and compact optoelectronic devices increases, substantial advancements in miniaturization are anticipated [6]. Two-dimensional (2D) materials hold significant potential in optoelectronic applications owing to their distinctive optical and electrical characteristics [7,8]. The intrinsic layered structure and remarkable structural stability of two-dimensional (2D) semiconductors enable them to preserve outstanding performance across different scales, rendering them highly promising for the advancement of next-generation optoelectronic devices. Several prototype photodetectors utilizing 2D materials have been experimentally and theoretically validated, demonstrating high responsivity, high detectivity, rapid reaction speed, customizable response spectrum, and robust stability [9]. In recent years, materials such as black phosphorus (BP), bismuth-based semiconductors, and emerging two-dimensional (2D) materials like GeH have attracted significant attention. These materials are characterized by their exceptional electrical transport properties, superior photoresponse capabilities, and notable air stability. Germanene is a two-dimensional material composed of a single layer of germanium atoms arranged in a honeycomb-like structure, similar to graphene but with a buckled configuration. Germanane (GeH), on the other hand, is a hydrogenated form of germanene. It is a one-atom-thick sheet of hydrogenated puckered germanium atoms, structurally similar to graphane. Germanane has a direct band gap of approximately 1.53 eV and exhibits high electron mobility, making it a promising candidate for various electronic and optoelectronic applications. These characteristics position it as a highly promising candidate for the development of high-performance photoelectrochemical (PEC) photodetectors and advanced optoelectronic devices [10,11,12,13]. Despite its advanced properties, GeH exhibits certain limitations. The valence band maximum (VBM) is predominantly composed of Ge 4p orbitals, while the conduction band minimum (CBM) involves a mixture of Ge 4s and 4p states. This overlap between the charge density distributions of the VBM and CBM increased the likelihood of photoexcited electrons recombining with holes, resulting in a high recombination rate that hinders the practical application of GeH [14]. In addition, the synthesis of most 2D GeH photodetectors has primarily relied on the topochemical deintercalation of the Zintl-phase CaGe_2_ in concentrated hydrochloric acid at low temperatures. This method leads to prolonged synthesis times, which hinder the large-scale production of devices and pose a considerable challenge to the advancement of high-performance optoelectronic devices [15]. To address these challenges, doping, surface modification, and nanostructure fabrication are potential strategies to modify the band gap, expand the spectral response, and enhance the photoelectric performance of 2D GeH photodetectors [16,17,18,19]. Selecting materials with significant energy band locations was crucial since they could function as collectors of photogenerated electrons and holes. This study has explored doping strategies to improve the PEC performance of GeH photoanodes, as doping not only modified the band gap and carrier concentration but also served as an effective method to tune the optical and optoelectronic properties of semiconductors [20,21,22]. Doping substitution with elements possessing higher orbital energies may have elevated the VBM and reduced the band gap [23]. Consequently, this study explored doping with elements from groups IIIA to IVA, specifically Te, as a means of improving the PEC performance of GeH photoanodes. Moderate Te doping, an underexplored approach, has been found to effectively enhance the electronic properties of GeH. Specifically, the alignment of Te’s conduction band edge with that of GeH promotes efficient charge separation. Meanwhile, the modified energy band structure lowers the interfacial kinetic barrier and reduces energy dissipation.

This study presents a high-performance self-powered photoelectrochemical (PEC) photodetector based on two-dimensional Te-GeH hybrid nanostructures, synthesized via in situ sintering and topochemical methods [24]. Te doping modified the VBM and CBM of GeH, enhancing carrier transfer and promoting charge separation, while effectively reducing charge recombination, which significantly boosts the photodetector’s electrical and optoelectronic performance. The photoresponse of the photodetector was optimized by varying the Te content, and its sensitivity and practical application were thoroughly evaluated under ideal conditions.

**Scheme 1:**
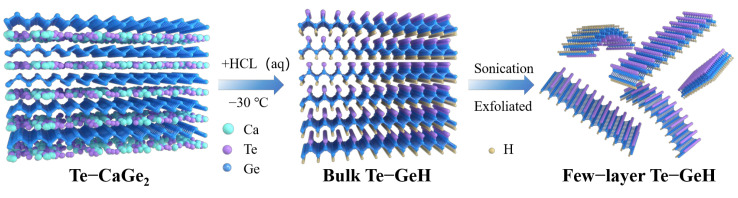
Synthesis scheme of Te-GeH.

Building upon existing synthesis methods, we successfully fabricated Te-GeH NSs through the doping of Te (Scheme 1). The resulting two-dimensional Te-GeH-based PEC photodetector exhibited a remarkable response rate of 708.5 μA/W, a detection rate of 2.13 × 10^9^ Jones, and ultrafast reaction times of 92 and 526 ms. With a small band gap of 1.32 eV, the Te-GeH photodetector demonstrated a broadband optical response ranging from ultraviolet (350 nm) to visible light (650 nm). Notably, it operated as a self-powered device without the need for an external bias, highlighting its potential for practical applications. This study highlighted the ability to control the operational characteristics of the PEC photodetector through external bias application and optimization of the electrolyte’s composition and concentration, further reinforcing the significant potential of Te-GeH NSs as a promising material platform for advancing next-generation optoelectronic devices.

## 2. Materials and Methods

### 2.1. Materials

Germanium (Ge, 200 mesh, 99.999%), calcium (Ca, 99.0%), anhydrous sodium sulfate (Na_2_SO_4_, 99.0%), and ethanol were acquired from Machlin (Shanghai Macklin Biochemical Co., Ltd., Shanghai, China). Tellurium (Te, ≥100 mesh, 99.999%) was acquired from Aladdin (Shanghai Aladdin Biochemical Technology Co., Ltd., Shanghai, China). Hydrochloric acid (HCl) was acquired from the Guangzhou Chemical Reagents Factory (Guangzhou, China). All chemical reagents were used as received without further purification, and deionized water (DI) was used exclusively throughout the experimental process. Indium tin oxide (ITO) glass (30 mm × 10 mm × 1 mm) with a sheet resistance below 6 Ω was procured from Shenzhen Huanan Xiangcheng Technology Co., Ltd. (Shenzhen, China).

### 2.2. Synthesis of 2D Te-GeH Nanostructures

Preparation of Zintl phase Te-CaGe_2_: In total, 1 g of germanium powder, 0.5 g of calcium granules, and varying concentrations of tellurium metal powder (0, 0.1, 0.3, 0.4, 0.5, and 1 g) were mixed and processed at ambient temperature for 30 min in an agate mortar. Upon meticulous grinding, we encapsulated the mixture in a bespoke vacuum quartz tube (180 mm × 10 mm; 3 mm wall thickness). The resultant quartz tube was vacuum-sealed and subjected to calcination in a muffle furnace using the following temperature profile: (1) increase temperature to 1000 °C at a rate of 4 °C/min; (2) keep at 1000 °C for 1440 min; (3) gradually cool to 500 °C at a rate of 2 °C/min followed by natural cooling to ambient temperature. Finally, to obtain high-quality Te-CaGe_2_ crystals, only the well-crystallized middle portion was extracted within a glove box.

Preparation of Te-GeH NSs with few layers: The CaGe_2_ and Te-CaGe_2_ solids were transferred to a round-bottom flask, where 30–40 mL of concentrated hydrochloric acid was added, and a vacuum was applied using a vacuum pump to establish a vacuum environment. Te-CaGe_2_ was agitated in strong hydrochloric acid at −30 °C for 48 h. The GeH product was purified by washing with DI water and subsequently with ethanol. The mixture was ultrasonic treated in ethanol solution at 200 W power for 30 min and kept in a 0 °C ice bath. The resultant solution was centrifuged at 1000 rpm for 10 min to exclude non-exfoliated Te-GeH. The supernatant containing Te-GeH NSs was carefully decanted and subsequently centrifuged at 10,000 rpm for 10 min to isolate the Te-GeH NSs. The resulting precipitate was collected and subsequently dried in a vacuum oven at 60 °C overnight for further use.

### 2.3. Fabrication and Photoresponse Characterization of the Photodetector

Fabrication of the PEC photodetector electrode: First, 1 mg of Te-GeH NSs was dispersed in 500 μL of ethanol and subjected to ultrasonication. The resulting low-layer Te-GeH NSs solution was subsequently spin-coated onto ITO conductive glass substrates. The coated ITO electrodes were first dried in a vacuum oven for 12 h and then stored at ambient temperature for subsequent use.

Preparation of PEC photodetector: The photochemical test was tested in a three-electrode system. The prepared Te-GeH was coated on ITO to form the working electrode. Platinum and saturated calomel electrodes served as counter and reference electrodes, respectively. Furthermore, 0.5 M Na_2_SO_4_ was employed as an electrolyte.

### 2.4. Materials Characterization

X-ray diffractometry (XRD, TD-3500, Dandong Tongda Technology Co., Ltd., Dandong, China) was conducted with Cu Kα radiation within a scan range of 5–80° (40 kV, 40 mA). The microstructures and compositions of the NSs were investigated via X-ray photoelectron spectroscopy (XPS, Thermo Scientific K-Alpha, Waltham, MA, USA). The morphology of various films and Te-GeH NSs was examined utilizing a field emission scanning electron microscope (SEM, Model SU8220, Hitachi High-Tech Corporation, Tokyo, Japan). The selected area electron diffraction (SAED) pattern and high-resolution transmission electron microscopy (HR-TEM, JEOL JEM-F200, Tokyo, Japan) were employed to examine the fine structures of the samples. Ultraviolet–visible diffuse reflectance spectroscopy (UV–vis DRS, Beijing Puriss General Instruments Co., Ltd., Beijing, China) was recorded using a TU-1901 spectrophotometer within an integrating sphere, covering a wavelength range from 250 to 800 nm. Raman spectra were obtained using an Xplora Plus Raman microscope (Xplora Plus, HORIBA France SAS, Palaiseau, France), which utilized a 532 nm laser as the excitation source.

### 2.5. Electrochemical Measurements

The optoelectronic characteristics of the Te-GeH NSs were evaluated using a photoelectrochemical testing system (PEC-2000, Beijing Perfectlight Technology Co., Ltd., Beijing, China). Amperometric current–time (*i*-*t*) curves and electrochemical impedance spectroscopy (EIS) measurements, covering a frequency range from 100 kHz to 0.01 Hz, were conducted with the CHI760E electrochemical workstation. A 50 W xenon arc lamp (PLS-FX300HU, Beijing Perfectlight Technology Co., Ltd., Beijing, China) was used as the light source, positioned 20 cm from the reaction vessel. To irradiate the photodetector, simulated sunlight (AM1.5G) and light at various wavelengths—350, 400, 450, 550, and 650 nm—were utilized, with the specific wavelengths selected using optical filters. In this study, the CHI760E electrochemical workstation (Shanghai Chenhua Instrument Co., Ltd., Shanghai, China) was employed to analyze the material. The resultant M-S curve was used to ascertain the material type, compare carrier concentration magnitudes, and identify the flat band position of the semiconductor.

## 3. Results and Discussion

The precursor Te-CaGe_2_ was first prepared by sintering at high temperature, and then intercalated with concentrated hydrochloric acid at low temperature (−30 °C) under vacuum environment to obtain two-dimensional Te-GeH. This method not only avoids the long low-temperature reaction process, but also realizes the regulation of the structure and property of the material by precisely controlling the stoichiometric ratio, as illustrated in Scheme 1. Finally, the liquid phase exfoliation method was employed to obtain few-layer Te-GeH NSs by sonication.

**Figure 1:**
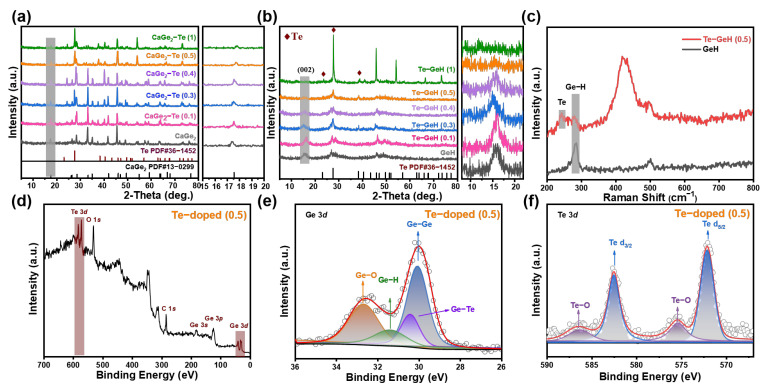
(**a**) XRD patterns of Te-CaGe_2_ and (**b**) Te-GeH. (**c**) Raman spectra of Te-GeH. (**d**) Survey XPS spectrum of Te-GeH. High-resolution XPS spectra of Te-GeH: (**e**) Ge 3, (**f**) Te 3d.

The structural configurations of Te-CaGe_2_ and Te-GeH were analyzed using X-ray diffraction (XRD), as depicted in Figure 1a,b. The XRD patterns indicated that the majority of the peaks correspond to the cubic phase of CaGe_2_ (PDF card No. 13-0299), with additional peaks ascribed to elemental Te (PDF card No. 36-1452). Doping may lead to lattice expansion, making the XRD characteristic peak lower. Notably, the intensity of the Te diffraction peak increased with doping concentration, and when the Te doping ratio exceeded 0.5, the distinct diffraction peak of GeH at the (002) crystal plane was significantly degraded, appearing as a broad scattering feature, indicative of substantial lattice disruption in GeH during the synthesis process [15]. To confirm the effective synthesis of Te-GeH, Raman spectroscopy was performed, which is a powerful tool for investigating the structural characteristics of two-dimensional materials. Raman spectra were acquired using a Raman microscope equipped with a 532 nm excitation laser under ambient conditions. As shown in Figure 1c, the E_2_ vibration of the Ge-H bond in GeH coincides with a faint second-order peak at 280 cm^−1^ in Te, overlapping at approximately 240 cm^−1^ in the Raman spectra [25,26]. In the exfoliated Te-GeH NSs, Te doping may change the distribution of electron clouds around Ge atoms, thus affecting the vibration characteristics of GE-H bonds. The change in electron cloud distribution will reduce the vibration frequency of the bond, resulting in Raman peak redshift [27]. Additionally, synchronized second-order absorption peaks at around 430 cm^−1^ and 500 cm^−1^ were detected. The survey spectrum in Figure 1d confirms the coexistence of Ge and Te elements in the Te-GeH samples. Figure 1e and Figure 1f present the high-resolution Ge 3d and Te 3d spectra, respectively. As shown in Figure 1e, the Ge 3d band exhibited four peaks: Ge-Ge at 29.84 eV, Ge-Te at 30.40 eV, Ge-H at 31.40 eV, and Ge-O at 32.68 eV [28,29]. The slight shift in the Ge 3d peak position was attributed to the lower electronegativity of Ge compared to Te, resulting in reduced electron affinity for the Ge atom. Te doping decreased the charge density around the Ge 3d atom while increasing it around the Te atom, which was reflected in the Te 3d spectrum (Figure 1f), where the peaks shifted toward lower binding energy. The peaks at 572.1 and 582.6 eV correspond to the 3d_5/2_ and 3d_3/2_ orbitals of Te 3d, while those at 575.5 and 586.6 eV were likely associated with Te-O bonds [25]. In the Te-GeH mid-valence band, Te 5p orbitals dominated, while the conduction band primarily comprised Ge 4s and 4p orbitals, thereby reducing the overlap of charge density distributions and preventing the recombination of photoexcited electrons into the valence band. Due to the difference in work function and electron affinity between GeH and Te, this interface can create a barrier for charge carriers. Modulating the height and configuration of the Schottky barriers could enhance electron transport and improve electronic device performance.

**Figure 2:**
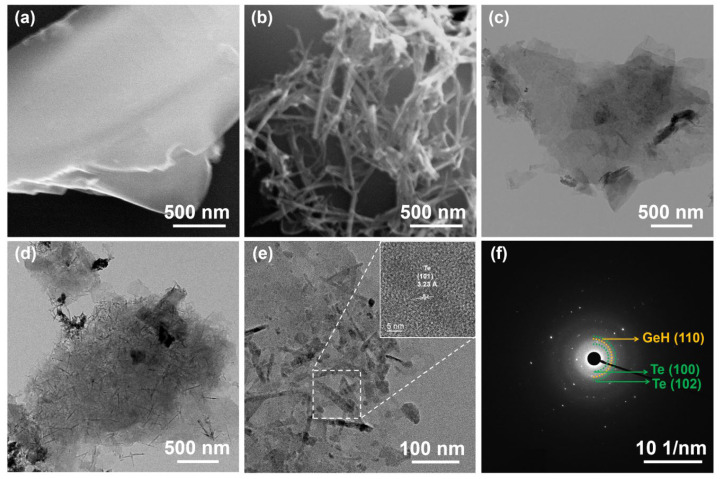
SEM images of (**a**) GeH and (**b**) Te-GeH. TEM images of (**c**) GeH and (**d**) Te-GeH. (**e**) HRTEM image and (**f**) SAED pattern for Te-GeH.

Figure 2a presents the SEM image of multiple layers of exfoliated GeH NSs exhibiting a full sheet-like morphology, which is consistent with the TEM shown in Figure 2c. The SEM images of Te-doped GeH NSs reveal a fibrous, cross-like structure on the surface (Figure 2b), which is further corroborated by the TEM image in Figure 2d. This fibrous cross-like structure was also observed when doped with varying ratios of Te (Figure S1a–d, Supporting Information). By integrating the findings from SEM and TEM, we observed that the material retains the inherent sheet-like morphological attributes of GeH [15] while exhibiting a highly interconnected fibrous cross-like architecture. We propose that this fibrous cross-topography forms a molecular-scale nanocomposite framework on the GeH sheet substrates. The fibrous structure was not isolated from the overall morphology; instead, it created a complex phase interface with the interpenetrating fiber cross-like arrangement of the nanostructures. We hypothesized that this unique morphology facilitates electron transport and enhances electrical conductivity, leading to efficient charge transport and collection. Additionally, the incorporation of nanostructures helps preserve the structural stability of the materials. The microstructure of Te-GeH NSs was analyzed using HR-TEM images and SAED patterns. The interplanar spacing of the stripes, associated with the crystal planes of each material, was determined using HR-TEM. As shown in Figure 2e, the measured d-spacing of 3.23 Å corresponds to the (101) planes of the Te structure [25] (inset of Figure 2e). The SAED pattern in Figure 2f illustrates the crystalline quality of the exfoliated Te-GeH NSs. The SAED pattern exhibited concentric rings, with the diffraction points signifying the polycrystalline characteristics of the sample [30], where the distinct green rings correspond to the (100) and (102) facets of Te [31], while the orange rings denote the (110) facets of GeH [26], aligning with the findings from the XRD (Figure 1a). The phase composition of Te-GeH was confirmed by XPS and Raman spectroscopy, thereby verifying the effective synthesis of Te-GeH. The XPS survey spectrum (Figure 1d) confirmed the coexistence of Ge and Te elements in the Te-GeH samples. The high-resolution Ge 3d and Te 3d spectra (Figure 1e,f) further elucidated the binding energies and chemical states of these elements, providing insights into the electronic structure and potential charge transfer mechanisms within the material.

**Scheme 2:**
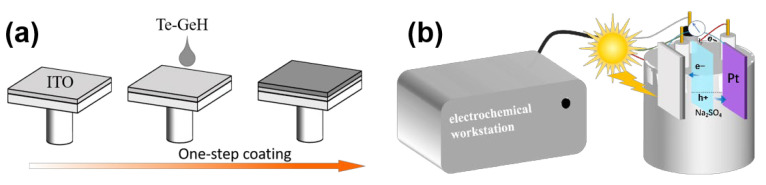
(**a**) Illustration of the spin-coating process for depositing Te-GeH nanostructures onto an ITO substrate. (**b**) Schematic diagram of electron transfer and exchange detected by an electrochemical workstation in the Te-GeH PEC photodetector under illumination.

Scheme 2a shows the fabrication of Te-GeH photoanodes by drip coating. As shown in Scheme 2b, in the Te-GeH PEC photodetector, upon illumination of the photoanode, the absorption of photons by Te-GeH leads to the excitation of electrons from the valence band to the conduction band, thereby generating electron (e^−^) and hole (h^+^) pairs. Under light exposure, the photogenerated electrons are driven by the built-in electric field to migrate from the conduction band of Te-GeH to the interface in contact with the electrolyte, and subsequently transfer to the ITO electrode. These electrons are then conveyed through an external circuit to the counter electrode (Pt electrode), resulting in the formation of an electric current. Concurrently, the photogenerated holes remain in the valence band of Te-GeH, accumulating at the material surface and participating in oxidation reactions. This photoelectrochemical effect enables the Te-GeH PEC photodetector to generate current under light conditions, thereby achieving efficient conversion of optical signals into electrical signals.

**Figure 3:**
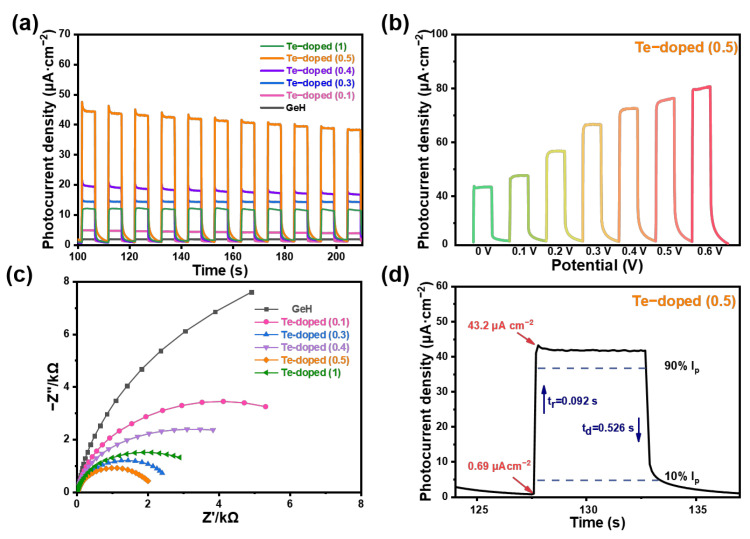
(**a**) The photocurrent density of various Te-GeH materials on ITO was measured under alternating on and off cycles, with a simulated light intensity of 60 mW cm^−2^ and zero bias potential. (**b**) The photocurrent density of the Te-GeH photodetector was evaluated under an external potential range of 0 to 0.6 V in 0.5 M Na_2_SO_4_. (**c**) Electrochemical impedance spectroscopy (EIS) of different Te-GeH NSs. (**d**) The calculated rise time (*t_r_*) and decay time (*t_d_*) of the photocurrent density.

The typical photoresponse behaviors (*i*-*t* curve) of Te-GeH demonstrated efficient photogenerated charge separation and migration across varying Te-GeH ratios, with the optimal performance observed at a Te-GeH ratio of 0.5, as shown in Figure 3a. The photocurrent density of the fabricated Te-GeH-based photodetector was analyzed under different bias potentials (Figure 3b), revealing commendable optoelectronic performance without the need for external voltage. As the bias voltage increases, the photocurrent density increases correspondingly, highlighting the ability to enhance device performance by adjusting the applied bias voltage. This enhancement in carrier transport and inhibition of recombination under external bias results in a stronger photoresponse. However, higher potentials also lead to more complex recombination processes involving photogenerated and externally driven carriers. This may result in a slightly longer time to reach a stable photocurrent. Electrochemical Impedance Spectroscopy (EIS) analysis indicated a significant reduction in load transfer resistance for the doped sample, suggesting improved conductivity and enhanced carrier movement within the material (Figure 3c). Te doping can enhance the electrical conductivity of GeH, thereby reducing the impedance of charge transport. When the doping concentration is 0.5, this improvement is most pronounced, as evidenced by the minimal impedance and optimal photoresponse performance. Excessively high Te doping concentrations may lead to intensified electrostatic interactions between ions, which in turn reduce ion mobility and increase the resistance to charge transport, resulting in higher impedance and diminished photoresponse performance. Te doping may also alter the internal structure and surface properties of GeH, thereby affecting the uniformity of charge distribution on the electrode surface. An appropriate doping concentration can optimize these characteristics, whereas either too high or too low a doping concentration may exert adverse effects. Additionally, the rise (*t_r_*) and decay (*t_d_*) times, defined as the intervals for the response to change from 10% to 90% [8], were measured at 92 and 526 ms as shown in Figure 3d, respectively, demonstrating significantly faster response times compared to pure GeH under identical conditions.

**Figure 4:**
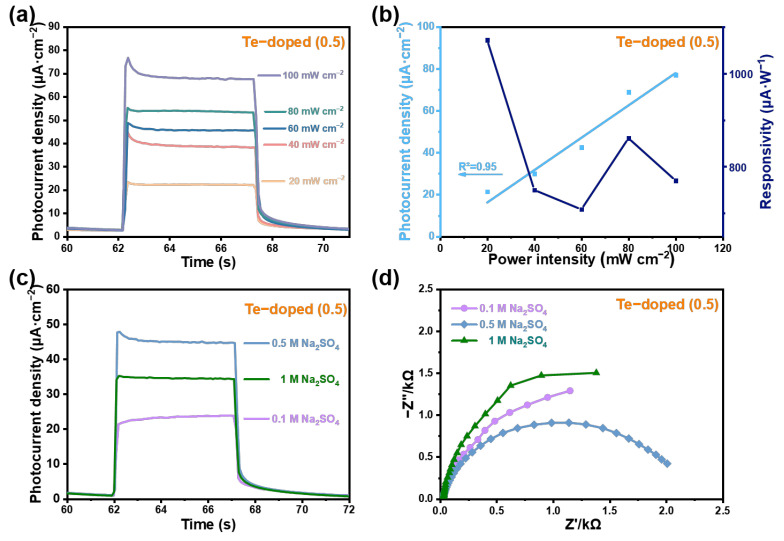
Photocurrent measurements of photodetectors based on Te-GeH NSs under varying conditions. (**a**) Photocurrent density of the Te-GeH photodetector under various power intensities (20 to 100 mW cm^−2^) at 0 V bias. (**b**) The corresponding photocurrent density and responsivity as a function of power intensity. (**c**) Photocurrent density of the Te-GeH photodetector in electrolytes of varying concentrations (0.1 M Na_2_SO_4_, 0.5 M Na_2_SO_4_, and 1 M Na_2_SO_4_) at 0 V. (**d**) EIS plots of Te-GeH NSs in the electrolytes of different concentrations.

The photoresponse characteristics of the PEC system were significantly affected by the intensity and wavelength of the incident light, as well as the applied bias, highlighting the need for further investigation. Figure 4a depicts the photocurrent density of the Te-GeH photodetector at varying light power intensities. To further explore the photoelectric properties of Te-GeH, we assessed its responsivity (R, μA·W^−1^) and specific detectivity (D*, Jones), as shown in Figure 4b. These two key parameters were defined by the following equations [6].

(1) $$R=\frac{I_{light}-I_{dark}}{P_{\lambda }S}$$

(2) $$D*=\frac{RS^{\frac{1}{2}}}{({2qI_{dark})}^{\frac{1}{2}}}$$

In this context, I_light_ and I_dark_ correspond to the currents measured under light exposure and in the absence of light, respectively. The power intensity of the incident light is represented by P_λ_, S denotes the effective illumination area, q refers to the electron charge, and I_dark_ indicates the current density under dark conditions. Figure 4b shows that the correlation between the calculated responsivity varied with illumination power density, where the Te-GeH-based photodetector achieved a responsivity of 708.5 μA/W at an irradiation power density of 60 mW cm^−2^, demonstrating the exceptional light detectability of the exfoliated Te-GeH-based PEC-type photodetector. This highlights its significant potential among various self-powered photodetectors. Table S3 further lists the values of responsivity and specific detectivity at different applied potentials and light intensities. In addition, Figure 4b illustrates the photocurrent density and photoelectric responsivity of Te-GeH photodetectors in response to varying irradiance power intensities, providing a deeper analysis of their photoresponse performance. The coefficient of determination R^2^ = 0.95 suggests that functionalized Te-GeH photoactive materials are capable of serving as photodetectors for quantifying sunlight radiation intensity. Furthermore, Figure 4c illustrates the photoresponse curves of the Te-GeH photodetector under illumination with a power intensity of 60 mW·cm^−2^ at a potential of 0 V in 0.1, 0.5, and 1 M Na_2_SO_4_ solutions. The photocurrent density exhibited a significant increase as the concentration of Na_2_SO_4_ rose from 0.1 M to 0.5 M, primarily due to the enhanced conductivity of the electrolyte. This enhancement in photocurrent density highlights the superior photoresponse characteristics of the Te-GeH photodetector in Na_2_SO_4_ solutions with moderate ion concentrations. However, at 1 M Na_2_SO_4_, the photocurrent density decreased, which is likely attributed to the increased electrostatic interactions between ions at higher concentrations. These interactions can reduce ion mobility and conductivity, as confirmed by the EIS diagram in Figure 4d. The 0.1 M Na_2_SO_4_ solution, with its relatively low ion concentration and number, exhibited poor electrical conductivity and high solution resistance. In general, higher solution concentrations typically lead to increased ion concentrations and improved conductivity, thereby reducing solution resistance. However, in the case of 1 M Na_2_SO_4_, the excessively high ion concentration may induce structural changes in the double layer, increasing the difficulty of charge transfer. This can be ascribed to the intensified electrostatic interactions between ions, which create a more complex and crowded environment. These interactions play a crucial role in determining ion mobility, as higher ion concentrations lead to stronger electrostatic correlations that impede ion movement. Consequently, the charge distribution on the electrode surface becomes non-uniform, and the dispersion effect is enhanced, resulting in increased impedance [32]. In contrast, the 0.5 M Na_2_SO_4_ solution demonstrated the smallest impedance, primarily due to its moderate ion concentration, which ensured low solution resistance, stable double layers on the electrode surface, and minimal dispersion effect. This optimal balance of ion concentration and conductivity in 0.5 M Na_2_SO_4_ solution is conducive to efficient charge transfer and enhanced photoresponse performance of the Te-GeH photodetector. To further investigate the changes in photoelectric performance, we also evaluated the photocurrent of Te-GeH photodetectors in acidic conditions (Figure S2).

**Figure 5:**
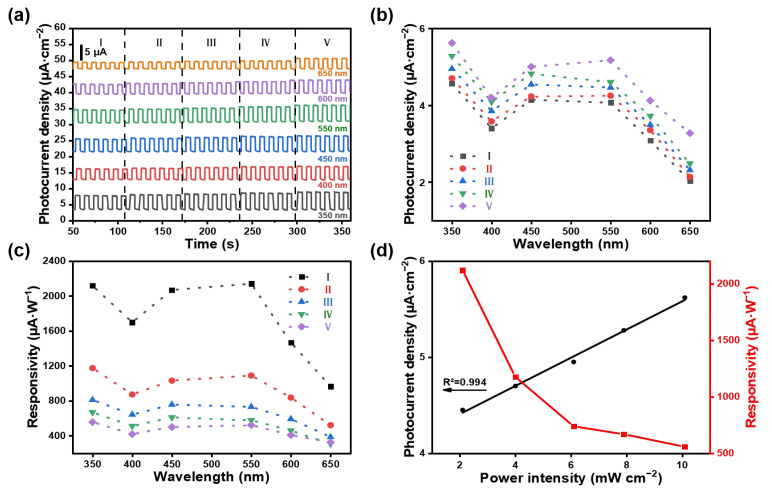
Photocurrent measurements of photodetectors based on Te-GeH NSs at various wavelengths. (**a**) The photocurrent density of Te-GeH-based photodetectors under different wavelengths. (**b**) The photocurrent density of the selected area (denoted as level V) was measured and plotted as a function of different incident wavelengths. (**c**) The responsivity plotted against different incident wavelengths. (**d**) Photocurrent density and responsivity as a function of different light power densities at 350 nm (level V). All tests were carried out in 0.5 M Na_2_SO_4_ electrolyte under 0 V bias.

Te-GeH exhibited a broad optical absorption spectrum ranging from 350 to 650 nm, as shown in the optical absorption data (Figure 5a). The spectral photoresponse characteristics of Te-GeH PEC photodetectors were evaluated under various wavelengths of light at power levels from I to V (refer to Tables S2–S4 in the Supporting Information for further details). Distinct on/off switching behaviors were observed under different wavelength irradiations, with a marked intensification at 350 nm. Figure 5b,c illustrates the photocurrent density and responsivity of the photodetector under 350 nm illumination, offering deeper insights into its photoresponse characteristics. When R^2^ = 0.994, it indicates that there is a high linear correlation between optical power and photocurrent. This means that in the studied optical power range, the photocurrent changes with the change in optical power almost in accordance with the linear law, indicating that the photodetector has good linear response characteristics under the light irradiation of 350 nm (Figure 5d). These findings indicate that the fabricated photodetector can be efficiently applied according to the specific wavelength of the incident light.

**Figure 6:**
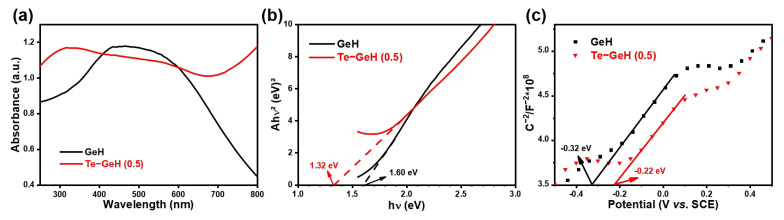
(**a**,**b**) UV-vis DRS absorption spectrum and the corresponding Tauc curve; (**c**) Mott–Schottky (M–S) plots of GeH and Te-GeH were measured at a frequency of 1.0 kHz in an aqueous solution of 0.5 M Na_2_SO_4_.

Figure 6a displayed the UV-vis DRS results, demonstrating that the Te-GeH NSs possessed a wide absorption spectrum across the UV-vis region. The associated Tauc plot (Figure 6b), generated from the absorption data, reveals an optical band gap of approximately 1.32 eV for the Te-GeH NSs, as calculated using the conventional Tauc method [33].

(3) $${\left(\alpha hv\right)}^{n}=K\left(hv-E_{g}\right)$$

The Tauc curve of Te-doped GeH showed a smaller optical band gap compared to undoped GeH, indicating a narrower band gap in the Te-GeH NSs. This reduced band gap suggests that 2D Te-GeH NSs may exhibit an extended photoresponse range, spanning from ultraviolet to visible light.

To investigate the underlying mechanisms responsible for the enhanced properties induced by Te doping, we examined the photogenerated carrier transfer kinetics of GeH both before and after Te doping. Mott–Schottky analyses were conducted as shown in Figure 6c, showing a positive slope for all samples, which is a hallmark of n-type semiconductors where electrons dominate as the primary charge carriers. A less steep slope in the Mott–Schottky plot indicates a higher carrier concentration, with Te-GeH demonstrating a significantly elevated charge carrier density [34]. The flat band potential (Vfb) was identified by the intersection of the Mott–Schottky plot with the *x*-axis. This characterization confirms the n-type nature of the material and provides insight into its band energy levels.

The interface band structures between Te and the matrix, highlighting the inter-face effects of the composites, are depicted in Scheme S1a. When two semiconductors with differing work functions and Fermi levels come into contact, band bending occurs. Electrons transfer from the semiconductor with a higher Fermi level to the one with a lower Fermi level until equilibrium is reached, aligning both Fermi levels (Scheme S1b). This spontaneous electron diffusion results in the creation of positive and negative charges at the phase interface, generating an internal electric field that induces either upward or downward band bending [35]. This process, which occurs during hybrid formation, significantly influences charge carrier movement within the hybrid, thereby impacting electron–hole recombination and separation. The internally generated electric field significantly improves the separation of photoexcited electron–hole pairs, thereby mitigating the rapid recombination of charge carriers. As a result, in hybrid-based devices, the photocurrent circulates multiple times throughout the carrier lifetime, thereby enhancing the photoresponse characteristics. Moreover, the work function and energy levels of the materials play a crucial role in carrier transfer kinetics. According to the literature and the flat band values derived from the Mott–Schottky analysis [12,31], it is evident that for n-type semiconductors, the conduction band (CB) is typically positioned at approximately −0.1 to −0.3 eV relative to the vacuum level [36]. Therefore, the energy level diagram presented in Scheme S1 underscores how doping alters the material’s band structure, facilitating charge migration to the surface and effectively modifying its electronic properties. This adjustment contributes to enhanced charge carrier dynamics within the hybrid system. The dissimilarity in the band structures of Te and GeH engenders the formation of a Schottky barrier at their contact interface, thereby inducing a built-in electric field. This built-in electric field effectively separates photogenerated carriers under light conditions, promoting electron and hole transport and reducing recombination, thus enhancing electrochemical charge transport. This structure significantly improves the photoelectric conversion efficiency of Te-GeH photoanode and contributes to the realization of efficient photovoltaic effect. This Schottky barrier enhances the separation of photogenerated carriers by creating a built-in electric field that drives electrons and holes apart, reducing recombination losses. The specific alignment of energy levels and the work functions of Te and GeH are critical in determining the efficiency of this charge separation process. The Te-doped GeH system, therefore, exhibits superior performance compared to undoped GeH and other nano-material-based PEC photodetectors, as demonstrated in Table 1.

## 4. Conclusions

In summary, the synthesis of Te-GeH NSs was achieved through a topochemical deintercalation approach using Te-CaGe_2_ as the precursor. The Te-GeH NSs demonstrated a broad light absorption across 250–800 nm, aligning with the spectral sensitivity of the PEC photodetector. Under zero-bias voltage and AM1.5G illumination, the Te-GeH-based photodetector exhibited excellent performance metrics with a responsivity of 708.5 μA/W and specific detectivity of 2.13 × 10^9^ Jones, alongside rapid response characteristics. The combination of facile synthesis and outstanding optoelectronic performance positions Te-GeH NSs as a promising material for high-performance, self-powered photodetectors suitable for UV-to-visible light applications.

## Figures and Tables

**Table 1:** The comparison of the Te-GeH-based PDs with the previously reported 2D-material-based PDs for PEC-type photodetectors.

Materials	Measurement Conditions	Rise/Decay Time (s)	Responsivity (μA W^−1^)	Ref.
Te-GeH	0.5 M Na_2_SO_4_, 0 V	0.092/0.526	708.5	This work
GeH	0.5 M Na_2_SO_4_, 0 V	0.24/0.74	22	[10]
Ga_x_Sn_1−x_Se	0.5 M KOH, 0.6 V	0.17/0.17	0.19	[37]
GeTe	0.3 M Na_2_SO_4_, 0.6 V	0.48/0.75	228.9	[38]
ReS_2_	1 M Na_2_SO_4_,0 V	5/4.96	2.15	[39]
Ti_2_CT*_x_*	0.5 M KOH, 0.8 V	2.23/6.13	18.1	[40]
Cr_2_Ge_2_Te_6_	0.5 M Na_2_SO_4_, 0 V	-	54.4	[41]
2D Te nanosheets	0.1 M KOH, 0.3 V	-	0.152	[25]
Co-CoO_x_/NC/Mo_2_CT*_x_*	0.5 M KOH, 0.3 V	0.4/0.4	13.13	[42]
SnS NSs	0.5 m Na_2_SO_4_, 0.4 V	0.6/0.3	18.2	[43]
g-C_3_N_4_@Bi	0.5 M KOH, 0 V	0.51/1.43	42.54	[44]
BP QDs-MoS_2_	1 M KOH, 0 V	0.051	15.7	[45]
borophene-OH	0.5 M KOH, 0 V	-	58.46	[46]
Sb NSs	0.5 M KOH, 0.6 V	0.9/1.3	1.6	[47]
2D Bi_2_S_3_	1 M KOH, 0 V	0.1/0.1	9	[48]
2D BP	0.1 M KOH, 0 V	0.5/1.4	2.2	[49]

## Data Availability

The data presented in this study are available upon request from the corresponding author.

## Supplementary Materials

The following supporting information can be downloaded at https://www.mdpi.com/article/10.3390/s25082530/s1, Figure S1. TEM images of Te-GeH (doped with different proportions of Te). Figure S2. Photoresponse behaviors of PDs based on Te-GeH NSs at acid conditions. (a) Photocurent density of Te-GeH photodetector under 60 mW cm^−2^ at 0 V. (b) EIS plots of Te-GeH NSs in 0.5 M H_2_SO_4_. Table S1. Photoresponse performance of Te-GeH NSs PDs: photocurrent density and responsivity under various light intensities. Table S2. The light powder densities of different wavelengths are used in this work. Table S3. The photocurrent density of the Te-GeH-based PDs for various wavelengths of light with different power intensities. Table S4. The responsivity of the Te-GeH-based PDs for various wavelengths of light with different power intensities. Scheme S1 (a) Schematic illustration of energy band diagrams of GeH and Te. (b) Proposed energy band of Te-GeH.

## References

[1] Zheng D., Pauporté T. (2024). Advances in Optical Imaging and Optical Communications Based on High-Quality Halide Perovskite Photodetectors. Adv. Funct. Mater..

[2] Fu J., Nie C., Sun F., Li G., Shi H., Wei X. (2024). Bionic Visual-Audio Photodetectors with In-Sensor Perception and Preprocessing. Sci. Adv..

[3] Demontis V., Durante O., Marongiu D., De Stefano S., Matta S., Simbula A., Ragazzo Capello C., Pennelli G., Quochi F., Saba M. (2024). Photoconduction in 2D Single-Crystal Hybrid Perovskites. Adv. Opt. Mater..

[4] Hou H.Y., Tian S., Ge H.R., De Chen J., Li Y.Q., Tang J.X. (2022). Recent Progress of Polarization-Sensitive Perovskite Photodetectors. Adv. Funct. Mater..

[5] Grätzel M. (2001). Photoelectrochemical Cells. Nature.

[6] Yang C., Wang G., Liu M., Yao F., Li H. (2021). Mechanism, Material, Design, and Implementation Principle of Two-Dimensional Material Photodetectors. Nanomaterials.

[7] Tao W., Kong N., Ji X., Zhang Y., Sharma A., Ouyang J., Qi B., Wang J., Xie N., Kang C. (2019). Emerging Two-Dimensional Monoelemental Materials (Xenes) for Biomedical Applications. Chem. Soc. Rev..

[8] Ye X., Li Y., Wei M., Yang Z., Li T., Chen C. (2025). Review: Photothermal Effect of Two-Dimensional Flexible Materials. J. Mater. Sci..

[9] Wang H., Song X., Li Z., Li D., Xu X., Chen Y., Liu P., Zhou X., Zhai T. (2024). Recent Advances in Two-Dimensional Photovoltaic Devices. J. Semicond..

[10] Liu N., Qiao H., Xu K., Xi Y., Ren L., Cheng N., Cui D., Qi X., Xu X., Hao W. (2020). Hydrogen Terminated Germanene for a Robust Self-Powered Flexible Photoelectrochemical Photodetector. Small.

[11] Han K., Huang G., Jia Y., Niu Q., Zheng Z., Wang B. (2024). Research on Photoelectrochemical Photodetectors Based on Bismuth 2D Thin Films. Opt. Mater..

[12] Zhu X., Cai Z., Wu Q., Wu J., Liu S., Chen X., Zhao Q. (2025). 2D Black Phosphorus Infrared Photodetectors. Laser Photon. Rev..

[13] Kumar A., Intonti K., Viscardi L., Durante O., Pelella A., Kharsah O., Sleziona S., Giubileo F., Martucciello N., Ciambelli P. (2024). Memory Effect and Coexistence of Negative and Positive Photoconductivity in Black Phosphorus Field Effect Transistor for Neuromorphic Vision Sensors. Mater. Horiz..

[14] Jin H., Dai Y., Ma X.-C., Yu L., Wei W., Huang B.-B. (2015). Enhancement of Photocatalytic Activity of a Two-Dimensional GeH/Graphene Heterobilayer Under Visible Light. RSC Adv..

[15] Bianco E., Butler S., Jiang S., Restrepo O.D., Windl W., Goldberger J.E. (2013). Stability and Exfoliation of Germanane: A Germanium Graphane Analogue. ACS Nano.

[16] Liang F., Chen W., Feng M., Huang Y., Liu J., Sun X., Zhan X., Sun Q., Wu Q., Yang H. (2021). Effect of Si Doping on the Performance of Gan Schottky Barrier Ultraviolet Photodetector Grown on Si Substrate. Photonics.

[17] Singh O.P., Sharma A., Gour K.S., Husale S., Singh V.N. (2016). Fast Switching Response of Na-Doped CZTS Photodetector from Visible to NIR Range. Sol. Energy Mater. Sol. Cells.

[18] Zhang C., Qian Q., Qin L., Zhu X., Wang C., Li X. (2018). Broadband Light Harvesting for Highly Efficient Hot-Electron Application Based on Conformal Metallic Nanorod Arrays. ACS Photonics.

[19] Yang T., Zheng B., Wang Z., Xu T., Pan C., Zou J., Zhang X., Qi Z., Liu H., Feng Y. (2017). Van der Waals Epitaxial Growth and Optoelectronics of Large-Scale WSe_2_/SnS_2_ Vertical Bilayer p–n Junctions. Nat. Commun..

[20] Dong J., Lian Y., Zhang Y., Perfetti L., Chen Z. (2025). Tuning the Band Gap in InSb (100) by Surface Chemical Doping. Appl. Surf. Sci..

[21] Feng D., Huang B., Li L., Li X., Gu Y., Hu W., Zhang Z. (2022). The Effects of Eu^3+^ Doping on the Epitaxial Growth and Photovoltaic Properties of BiFeO_3_ Thin Films. J. Mater. Sci. Technol..

[22] Shkir M., Ashraf I.M., AlFaify S., El-Toni A.M., Ahmed M., Khan A. (2020). A Noticeable Effect of Pr Doping on Key Optoelectrical Properties of CdS Thin Films Prepared Using Spray Pyrolysis Technique for High-Performance Photodetector Applications. Ceram. Int..

[23] Chen S.H., Ho C.M., Chang Y.H., Lee K.M., Wu M.C. (2022). Efficient Perovskite Solar Cells with Low J-V Hysteretic Behavior Based on Mesoporous Sn-Doped TiO_2_ Electron Extraction Layer. Chem. Eng. J..

[24] Xiao X., Wang H., Urbankowski P., Gogotsi Y. (2018). Topochemical Synthesis of 2D Materials. Chem. Soc. Rev..

[25] Xie Z., Xing C., Huang W., Fan T., Li Z., Zhao J., Xiang Y., Guo Z., Li J., Yang Z. (2018). Ultrathin 2D Nonlayered Tellurium Nanosheets: Facile Liquid-Phase Exfoliation, Characterization, and Photoresponse with High Performance and Enhanced Stability. Adv. Funct. Mater..

[26] Giousis T., Potsi G., Kouloumpis A., Spyrou K., Georgantas Y., Chalmpes N., Dimos K., Antoniou M.K., Papavassiliou G., Bourlinos A.B. (2021). Synthesis of 2D Germanane (GeH): A New, Fast, and Facile Approach. Angew. Chemie Int. Ed..

[27] Husain S., Alkhtaby L.A., Bhat I., Giorgetti E., Zoppi A., Muniz Miranda M. (2014). Study of Cobalt Doping on Structural and Luminescence Properties of Nanocrystalline ZnO. J. Lumin..

[28] Hartman T., Šturala J., Luxa J., Sofer Z. (2020). Chemistry of Germanene: Surface Modification of Germanane Using Alkyl Halides. ACS Nano.

[29] Shalvoy R.B., Fisher G.B., Stiles P.J. (1977). Bond Ionicity and Structural Stability of Some Average-Valence-Five Materials Studied by X-Ray Photoemission. Phys. Rev. B.

[30] Nwankwo M.C., Ezealigo B., Nwanya A.C., Nkele A.C., Agbogu A., Chime U., Asogwa P.U., Ezekoye B.A., Ekwealor A.B.C., Osuji R.U. (2020). Syntheses and Characterizations of GO/Mn_3_O_4_ Nanocomposite Film Electrode Materials for Supercapacitor Applications. Inorg. Chem. Commun..

[31] Li J., Zhang J., Chu J., Yang L., Zhao X., Zhang Y., Liu T., Lu Y., Chen C., Hou X. (2023). Tailoring the Epitaxial Growth of Oriented Te Nanoribbon Arrays. iScience.

[32] Parida S., Das S., Mallik A. (2024). Application of Electrochemical Impedance Spectroscopy (EIS) to Study the Effect of Temperature and Ion Concentration During Electroplating of Copper from an Acidic Bath. Trans. Indian Inst. Met..

[33] Tauc J. (1968). Optical Properties and Electronic Structure of Amorphous Ge and Si. Mater. Res. Bull..

[34] Barnes P., Zuo Y., Dixon K., Hou D., Lee S., Ma Z., Connell J.G., Zhou H., Deng C., Smith K. (2022). Electrochemically Induced Amorphous-to-Rock-Salt Phase Transformation in Niobium Oxide Electrode for Li-Ion Batteries. Nat. Mater..

[35] Zhang P., Zhao S., Wang H., Zhang J., Shi J., Wang H., Yan D. (2017). Relation Between Interfacial Band-Bending and Electronic Properties in Organic Semiconductor Pentacene. Adv. Electron. Mater..

[36] Chun W.J., Ishikawa A., Fujisawa H., Takata T., Kondo J.N., Hara M., Kawai M., Matsumoto Y., Domen K. (2003). Conduction and Valence Band Positions of Ta_2_O_5_, TaOn, and Ta_3_N_5_ by UPS and Electrochemical Methods. J. Phys. Chem. B.

[37] Liang Z., Hao R., Luo H., He Z., Su L., Fan X. (2024). Enhancing the Photo-Response Performance of a SnSe-Based Photoelectrochemical Photodetector via Ga Doping. J. Mater. Chem. C.

[38] Wang H., Lu C., Dong W., Xue X., Li E., Zhao Q., Xu X. (2024). Photoelectrochemical Photodetector Based on Germanium Telluride Film Synthesized by Physical Vapor Deposition. ACS Appl. Nano Mater..

[39] Zhang R., Wang K., Li J. (2023). A Robust 3D Self-Powered Photoelectrochemical Type Photodetector Based on ReS_2_ Nanoflowers. J. Mater. Sci. Mater. Electron..

[40] Yu R., Qiao H., Zhou Y., Liao G., Huang Z., Wang Z., Qi X. (2023). Etching Exfoliated Ti_2_CT*x* Nanosheets for Photoelectrochemical Photodetectors with Enhanced Performance and Alkaline Stability. J. Electron. Mater..

[41] Deng J., Qiao H., Luo S., Zhang S., Huang Z., Wang Z., Qi X. (2023). Simulation Photosynthesis Improves the Photoresponse Performance of Cr_2_Ge_2_Te_6_ Nanosheets for High-Performance Self-Powered Photoelectrochemical Photodetectors. ACS Appl. Nano Mater..

[42] Luo S., Wu Z., Zhao J., Luo Z., Qiu Q., Li Z., Wu H., Xing G., Wu C. (2022). ZIF-67 Derivative Decorated MXene for a Highly Integrated Flexible Self-Powered Photodetector. ACS Appl. Mater. Interfaces.

[43] Xu X., Bai X., Han T., Dong W., Zhang Y., Wang Y., Lu C., Hua D. (2022). High Performance UV–Vis Photodetectors Based on Tin Monosulfide Film Synthesized by Physical Vapor Deposition. Appl. Surf. Sci..

[44] Zhang Y., Xu Y., Guo J., Zhang X., Liu X., Fu Y., Zhang F., Ma C., Shi Z., Cao R. (2021). Designing of 0D/2D Mixed-Dimensional van der Waals Heterojunction over Ultrathin g-C_3_N_4_ for High-Performance Flexible Self-Powered Photodetector. Chem. Eng. J..

[45] Qiao H., Li Z., Huang Z., Ren X., Kang J., Qiu M., Liu Y., Qi X., Zhong J., Zhang H. (2020). Self-Powered Photodetectors Based on 0D/2D Mixed Dimensional Heterojunction with Black Phosphorus Quantum Dots as Hole Accepters. Appl. Mater. Today.

[46] Wang X., Liang J., You Q., Zhu J., Fang F., Xiang Y., Song J. (2020). Bandgap Engineering of Hydroxy-Functionalized Borophene for Superior Photo-Electrochemical Performance. Angew. Chemie Int. Ed..

[47] Su L., Tang X., Fan X., Ma D., Liang W., Li Y., Zhang H. (2019). Halogenated Antimonene: One-Step Synthesis, Structural Simulation, Tunable Electronic and Photoresponse Property. Adv. Funct. Mater..

[48] Huang W., Xing C., Wang Y., Li Z., Wu L., Ma D., Dai X., Xiang Y., Li J., Fan D. (2018). Facile Fabrication and Characterization of Two-Dimensional Bismuth(Iii) Sulfide Nanosheets for High-Performance Photodetector Applications under Ambient Conditions. Nanoscale.

[49] Ren X., Li Z., Huang Z., Sang D., Qiao H., Qi X., Li J., Zhong J., Zhang H. (2017). Environmentally Robust Black Phosphorus Nanosheets in Solution: Application for Self-Powered Photodetector. Adv. Funct. Mater..

