# Supplementary material for: High-Performance Self-Powered Photodetector Enabled by Te-Doped GeH Nanostructures Engineering
Source: Sensors (Basel). 2025 Apr 17;25(8):2530. doi: 10.3390/s25082530 (PMC12030971; doi:10.3390/s25082530)
Supplement: Supplementary file 1 [file sensors-25-02530-s001.zip › sensors-3565666-supplementary.pdf]

sensors

MDPI

Supporting Information for

High-Performance Self-Powered Photodetector Enabled by Te-Doped GeH Nanostructures Engineering

Junting Zhang [1], Jiexin Chen [1], Shuojia Zheng [1], Da Zhang [1], Shaojuan Luo [1,2,3,*] and Huixia Luo [2]

[1] School of Chemical Engineering and Light Industry, Guangdong University of Technology, Guangzhou 510006, China; zjunting2023@126.com (J.Z.); chenjiexin0501@163.com (J.C.); shuo2959731365@163.com (S.Z.); dazhang102000@163.com (D.Z.)
[2] State Key Laboratory of Optoelectronic Materials and Technologies, Sun Yat-sen University, Guangzhou 510275, China; luohx7@mail.sysu.edu.cn
[3] Jieyang Branch of Chemistry and Chemical Engineering Guangdong Laboratory, Jieyang 515200, China
* Correspondence: kesjluo@gdut.edu.cn

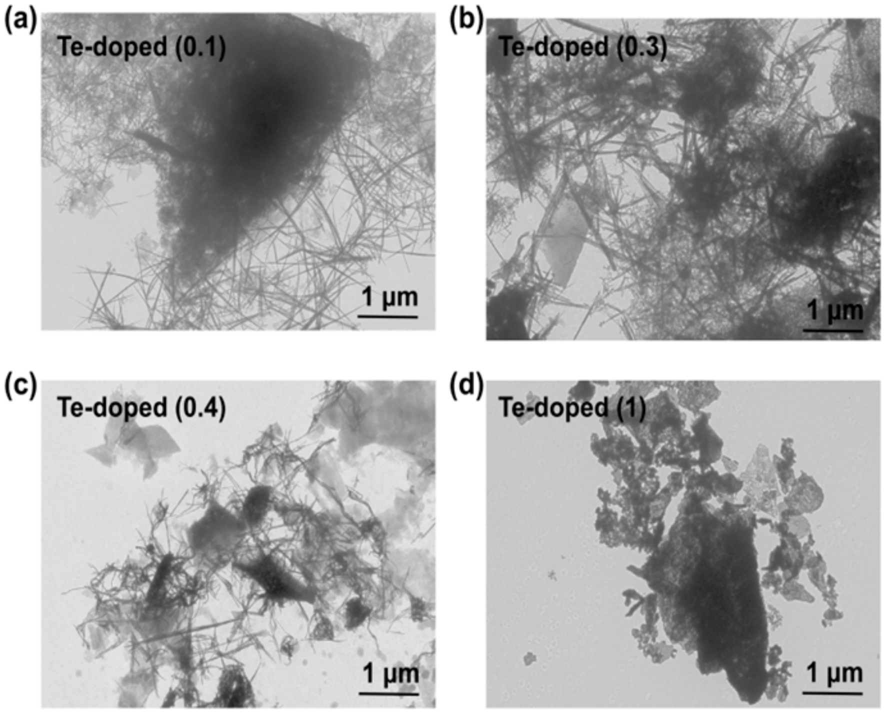

Figure S1. TEM images of Te-GeH (doped with different proportions of Te) .

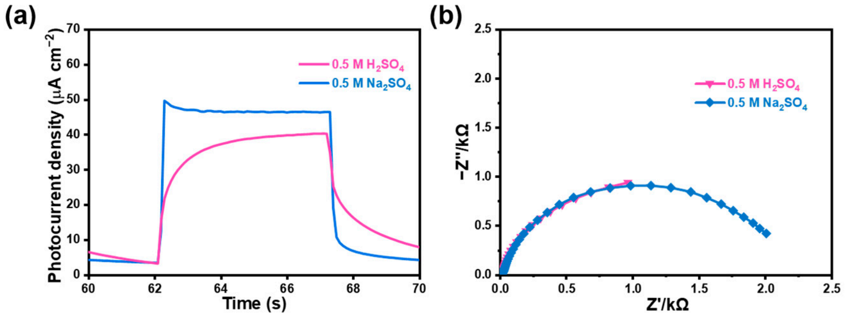

Figure S2. Photoresponse behaviors of PDs based on Te-GeH NSs at acid conditions. (**a**) Photocurent density of Te-GeH photodetector under 60 mW cm^{-2} at 0 V. (**b**) EIS plots of Te-GeH NSs in 0.5 M H_2SO_4.

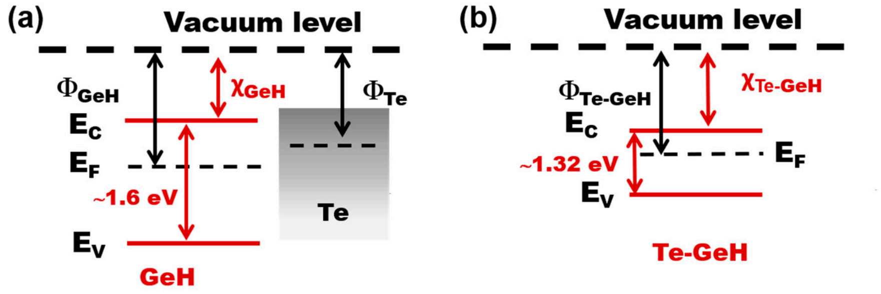

Scheme S1 (**a**) Schematic illustration of energy band diagrams of GeH and Te. (**b**) Proposed energy band of Te-GeH.

Table S1. Photoresponse performance of Te-GeH NSs PDs: photocurrent density and responsivity under various light intensities.

Power intensity ($mW\cdot cm^{-2}$)	Photocurrent density （$\mu A\cdot cm^{-2}$)	Responsivity ($\mu A\cdot W^{-1}$)
20	21.43	1071.50
40	31.23	780.73
60	42.51	708.50
80	68.88	861.00
100	77.00	770.05

Table S2. The light powder densities of different wavelengths are used in this work.

	P ($mW\cdot cm^{-2}$)				
**Wavelength**	**I**	**II**	**III**	**IV**	**V**
**350 nm**	2.1	4.0	6.1	7.9	10.1
**400 nm**	2.0	4.1	6.0	8.0	10.0
**450 nm**	2.0	4.1	6.0	7.9	10.0
**550 nm**	1.9	3.9	6.1	8.1	9.9
**600 nm**	2.1	4.0	5.9	8.1	10.1
**650 nm**	2.1	4.1	6.0	8.1	10.1

Table S3. The photocurrent density of the Te-GeH-based PDs for various wavelengths of light with different power intensities.

	Photocurrent density （$\mu A\cdot cm^{-2}$）				
**Wavelength**	**I**	**II**	**III**	**IV**	**V**
**350 nm**	4.45	4.70	4.95	5.28	5.62
**400 nm**	3.40	3.58	3.85	4.09	4.19
**450 nm**	4.14	4.23	4.54	4.82	5.00
**550 nm**	4.07	4.25	4.46	4.68	5.17
**600 nm**	3.08	3.35	3.49	3.72	4.12
**650 nm**	2.03	2.13	2.31	2.48	3.27

Table S4. The responsivity of the Te-GeH-based PDs for various wavelengths of light with different power intensities.

	Responsivity （$\mu A \cdot W^{-1}$）				
**Wavelength**	**I**	**II**	**III**	**IV**	**V**
**350 nm**	2119.1	1175	811.5	668.4	556.4
**400 nm**	1699.5	873.2	641.7	511.3	419
**450 nm**	2070	1031.7	756.7	610.1	500
**550 nm**	2142.1	1089.7	731.1	577.8	522.2
**600 nm**	1466.7	837.5	591.5	459.3	407.9
**650 nm**	965.7	520.4	385.5	306.4	324.1
